# Robot-assisted radical cystectomy and ileal conduit with Hugo™ RAS system: feasibility, setting and perioperative outcomes

**DOI:** 10.1590/S1677-5538.IBJU.2023.0349

**Published:** 2024-02-07

**Authors:** Josep Maria Gaya, Alessandro Uleri, Isabel Sanz, Giuseppe Basile, Paolo Verri, Pedro Hernandez, Angelo Territo, Oscar Rodríguez Faba, Andrea Gallioli, Alberto Breda, Joan Palou

**Affiliations:** 1 Autonomous University of Barcelona Department of Urology Fundació Puigvert Barcelona Spain Department of Urology, Fundació Puigvert, Autonomous University of Barcelona, Barcelona, Spain

## Abstract

**Introduction:**

Robotic approach has shown its feasibility and safety with respect to open approach for radical cystectomy (1). The performances of Hugo™ RAS system (Medtronic, Minneapolis, USA) have been demonstrated in several clinical scenarios (2-5). We report the feasibility and surgical settings of the first series of robot-assisted radical cystectomy (RARC) with intracorporeal ileal-conduit performed with Hugo™ RAS system.

**Methods:**

Two patients were submitted to RARC with ileal conduit at our institution. The trocar placement scheme and the operating room setting with docking angles of the four arms were already described (6). A 12-mm and a 5-mm trocar for the assistant were placed. In both cases, an ileal-conduit with a Wallace type-1 uretero-enteric derivation was performed intra-corporeally.

**Results:**

The first patient was a 71-year-old male with a very-high risk non-muscle invasive bladder cancer(BC), and the second patient was a 64-year-old male with a diagnosis of T2 high-grade BC. Operative times were 360 and 420 minutes with a docking time of 12 and 9 minutes, respectively. No intraoperative complications occurred. The estimated blood loss was 200ml and 400ml, respectively. The second patient developed an ileus on postoperative day 4 (Clavien-Dindo grade 2). No positive surgical margins were recorded. No recurrence nor progression occurred during follow-up.

**Conclusion:**

RARC with intracorporeal ileal conduit urinary diversion is feasible with Hugo™ RAS system. We provided insight into the surgical setting using this novel robotic platform to help new adopters to face this challenging procedure. These findings may help a wider distribution of robotic programs for BC treatment.

**Available at:** http://www.intbrazjurol.com.br/video-section/20230349_Uleri_et_al

**Int Braz J Urol. 2023; 49 (Video #15): 787-8**
